# The Awareness of the Role of Commercial Determinants of Health and the Readiness to Accept Restrictions on Unhealthy Food Advertising in Polish Society

**DOI:** 10.3390/nu15224743

**Published:** 2023-11-10

**Authors:** Urszula Zwierczyk, Mateusz Kobryn, Mariusz Duplaga

**Affiliations:** Department of Health Promotion and e-Health, Institute of Public Health, Faculty of Health Sciences, Jagiellonian University Medical College, Skawińska Str. 8, 31-066 Krakow, Poland; urszula.zwierczyk@uj.edu.pl (U.Z.); m.kobryn@doctoral.uj.edu.pl (M.K.)

**Keywords:** commercial determinants of health, health literacy, food literacy, e-health literacy

## Abstract

The negative consequences of commercial determinants of health (CDoH) have become a major challenge for public health systems, especially in terms of non-communicable diseases (NCDs). CDoH are defined as profit-driven factors that influence health. In this study, we assessed the awareness of CDoH and the attitudes toward potential restrictions on advertising, as well as fiscal interventions targeting food products with harmful effects on health in Polish society. Our analysis is based on data from a computer-based web interviewing (CAWI) survey performed in May 2022 among 2008 adult internet users from Poland. Multivariable logistic regression models were developed for variables derived from three items exploring the respondents’ understanding of the relationship between CDoH and NCDs, as well as three items asking about their acceptance of a prohibition of advertising unhealthy products during sports events, a general ban on unhealthy food advertising, and their attitudes toward sugar-sweetened beverages (SSBs). Food (FL) and e-health literacy (eHL) levels were consistently positive predictors of both awareness of CDoH and acceptance of the proposed actions. Both higher FL and eHL were significantly associated with the opinion that advertising unhealthy food is associated with the prevalence of NCDs (OR, 95% CI: 1.03, 1.02–1.05, and 1.04, 1.02–1.06, respectively). Health literacy was less frequently a significant predictor of the dependent variables. Among sociodemographic factors, a respondent’s level of education and age showed a significant relationship with their awareness and acceptance of countermeasures against CDoH. Respondents with a university master’s level of education were more likely to agree with the statement on the relationship between big industry profits and harm to society’s health (OR, 95% CI: 1.96, 1.42–2.69) and to support a ban on advertising unhealthy food similar to that for tobacco products (OR, 95% CI: 1.66, 1.21–2.27). Respondents suffering from chronic diseases were also consistently more likely to show a greater understanding of the harmful impact of CDoH and support proposed restrictions. For example, they were more likely to agree with restrictions on advertising harmful products during sports events (OR, 95% CI: 1.23, 1.02–1.50) and the introduction of a sugar tax (OR, 95% CI: 1.26, 1.03–1.54). Our study revealed that more than 50% of the Polish population is conscious of the problem of the harmful effects of big industries producing and selling processed food, sugar-sweetened beverages, and alcoholic beverages. Interestingly, slightly more than half of the respondents supported the introduction of restrictions on advertising such products. Still, only approximately 30% of them accepted a sugar tax to counter the obesity epidemic. The results of our study indicate that Polish society is open to the introduction of regulations aimed at limiting the impact of commercial determinants of health. To our knowledge, this is one of the first studies to assess the awareness of CDoH and the acceptance of restrictions to limit their impact.

## 1. Introduction

Harmful health behaviors, including increased consumption of products with a high content of saturated fats, sugar, and salt, the use of tobacco products and alcoholic beverages, and limited physical activity, are indicated as the key risk factors for noncommunicable diseases (NCDs) [1]. Health promotion and disease prevention interventions targeting the risk factors of NCDs usually emphasize the role of appropriately shaping lifestyle, frequently exaggerating the importance of personal responsibility [2]. This happens even though the role of social determinants of health is widely understood and commented on [3,4]. However, it is known that attributing unfavorable health behaviors to individual responsibility only is a substantial simplification [5]. In most cases, health behaviors are not only the result of individual attitudes and choices but are also the result of the wider socioeconomic environment [6].

Today, it is obvious that the spectrum of social determinants that go far beyond personal responsibility should be extended to include commercial determinants of health (CDoH). Ten years ago, multinational companies active in the processed food, alcohol, and tobacco industries were already being called ‘vectors’ of the NCD epidemic [1]. The role of these vectors in the transmission of diseases relies not only on the manufacture, sale, and marketing of products that are harmful to health but also on the immense influence of these industries on legislation and other policies [7].

The harmful influence of big industries, e.g., Big Tobacco, on the population’s health has been clearly evident since at least the first half of the 20th century [8,9]. However, CDoH have only recently begun to take a prominent place in integrated models of health determinants [10]. Throughout the last ten years, epidemiologists have emphasized the importance of CDoH, pointing to the links between the NCD pandemic and the unbridled pursuit of profit manifested by multinational corporations [1], and public health specialists have been unmasking the mechanisms of commercial influences [11].

CDoH can be defined as profit-driven factors that influence health [12]. However, considering CDoH in the context of harmful health impacts, Kickbush et al. [13] proposed another definition, describing CDoH as “strategies and approaches used by the private sector to promote products and choices that are detrimental to health” [13]. Some other authors have underlined that there is a conflict between the goods and services understood as CDoH and the aim of public health [14]. According to Lencucha and Thow (2019), the activities of the industries that harm health are currently one of the major problems for public health [15].

CDoH may be perceived from a macro-level (global consumer society) and a micro-level (consumer health behaviors) perspective. The macro-level factors identified by de Lacy-Vawdon and Liviginstone include the economic and political power exerted by large corporations and social constructs such as neoliberalism, capitalism, globalization, trade agreements, corporate structures and rights, and even regulations [16]. The main activities through which CDoH manifest include marketing, corporate political activities (e.g., lobbying), litigation, political donations, integrated supply chains, and finally, products and production that harm health [16].

Mialon distinguished three areas of CDoH: (1) unhealthy commodities, (2) business, market, and political practices used to sell these commodities and secure a favorable policy environment, and finally, (3) global drivers of ill-health, including market-driven economies and globalization, facilitating the development of such practices [17].

The marketing and advertising practices of unhealthy commodities are most frequently addressed in the context of CDoH, especially when they concern children and youth as the target audience. The industries most often discussed as sources of CDoH in the literature include those supplying food, alcohol, and tobacco. Less frequently, the pharmaceutical or automotive industry is mentioned. These industries have developed sophisticated methods of attracting consumers and obstructing behavioral changes, e.g., reducing the use of unhealthy commodities. Petticrew et al. described examples of such methods as “dark nudges” and “sludge” in the case of the alcohol industry [18]. These authors underlined how dark nudges exploit cognitive biases to promote misinformation and hide accurate information. The long-standing relationship between worldwide sports organizations and CDoH-producing industries, which results in unhealthy commodities commonly being advertised during sporting events, may be another example of such strategies [19,20,21].

Lee and Crosbie [22] discussed CDoH through the lens of structure and agency [22]. In social sciences, agency is used in relation to the individual and collective capacity to make decisions and act independently. In turn, structure denotes the framework within which human agency occurs [23]. Lee and Crosbie (2020) believe that the main question that should be asked concerning CDoH is what must be undertaken to address corporate agency [22].

CDoH may be viewed as both proximal and distal determinants. Usually, proximal determinants of health are those that directly impact disease. Distal factors are associated with socioeconomic determinants and influence proximal risk factors [24].

Among the methods applied to measure the impact of CDoH in the context of public health, only a few tools have been proposed and developed. Baum et al. developed a corporate health impact assessment approach that employs mixed methods and techniques, including document analysis, media analysis, semi-structured interviews, and geographic information systems [25]. Another team developed a corporate permeation index (CPI), defined as the extent to which corporations are embedded in a country’s political, legal, social, economic, and cultural fabric [26]. The authors applied 25 indicators showing corporate practices that impact population health.

The framework proposed by Lee et al. is based on these two approaches [24]. They developed their framework for measuring CDoH exposure based on two agency components (market and nonmarket strategies) and four structural components, including political and economic systems, stratification, governance, and norms. The choice of indicators for every element depends on the available data. For example, market strategies can be assessed with the indicator showing per capita spending to advertise health-harming products. Based on the values of the applied indicators, the authors of the framework could assess the risk exposures in terms of agency and structural influences per country. This framework may be useful in evaluating the country-level influence of CDoH; however, it is less useful for assessing individual agency following the nomenclature mentioned above.

Unexpectedly, the awareness of the importance of CDoH within societies has been studied much less. We believe that without society’s understanding and awareness of the role of CDoH, public health systems will not be able to adopt effective prevention measures in this domain. Excluding the advertising of unhealthy commodities from sporting events could be an example of such a measure requiring a wide consensus [20]. It also seems that public health authorities would require strong support from society to introduce more stringent regulations on the nutritional quality, price, and marketing of food products [27].

This study’s main aim was to assess society’s perception of the role of the big food industry in determining individuals’ health. Specifically, we wanted to determine if respondents are aware of the influence of advertising and other marketing practices in the increased consumption of unhealthy food. Furthermore, we asked respondents whether they see a relationship between large corporations earning high profits by offering highly processed food and the prevalence of NCDs. Finally, we evaluated to what extent respondents would accept restrictions on advertising unhealthy products and if they perceive fiscal interventions like a ‘sugar tax’ as an appropriate measure to limit the risk of NCDs, as exemplified by obesity. We also analyzed the role of sociodemographic factors and various types of health literacy as determinants of the described attitudes.

## 2. Materials and Methods

### 2.1. Survey

The data analyzed in this study were obtained from a computer-assisted web Interviewing (CAWI) survey on a representative sample of 2008 adult Polish internet users. The survey was executed in May 2022. Assuming that the relevant population was approximately 28,600,000 [28], with the sample size of 2008 respondents, the sampling error was approximately 2.2%, assuming a fraction of 0.5 and a confidence level of 0.95. The survey was performed by the PBS Company [29], a market and opinion research company selected as a result of the bidding procedure obligatory at the university. The study sample was generated from an Internet panel conducted by the PBS Company [30] to reflect the structure of the population of Internet users in Poland in terms of age, gender, education, place of residence, and territorial unit [28]. The inclusion criteria included an age of at least 18 years and the use of the Internet.

This study received a positive opinion from the Bioethical Committee at the Jagiellonian University in Krakow, Poland (decision no. 1072.6120.197.2021 issued on 29 September 2021, with further amendments). The invited participants on the PBS panel were provided with information about this study’s aims and then asked to provide informed consent before completing the questionnaire. Respondents could withdraw from the survey at any moment. This study’s authors received anonymized data from the PBS Company without any personal data that would enable the identification of respondents.

### 2.2. Questionnaire

The questionnaire utilized in the survey consisted of 86 individual items. The assessment of the awareness of CDoH and the acceptance of restrictions on their advertising was based on six items established based on the relevant literature [31,32,33]. The questionnaire comprised several validated tools, including the 10-item e-Health Literacy Scale (eHEALS) [34,35], the 6-item European Health Literacy Survey Questionnaire (HLS-EU-Q6) [36,37], the 11-item Short Food Literacy Questionnaire (SFLQ) [38,39], and the 23-item Eating Motivation Scale (EATMOT) [40]. Furthermore, items asking about the consumption of selected foods, health behaviors, the use of the Internet, and sociodemographic and economic characteristics were included in the questionnaire. In this analysis, the data originating from items exploring the awareness of the health effects of CDoH and the attitudes to restrictions seeking to limit these effects, measurements of health literacies, and items asking about the sociodemographic and economic characteristics of respondents were used.

### 2.3. Measures

The awareness of CDoH was based on three items asking for the respondents’ opinions about the following:

- The relationship between profits achieved by large corporations by selling products with a high sugar content, sweetened beverages, highly processed food, and fast foods, and the health status of societies.
- The influence of advertising highly processed food on purchasing decisions.
- The association between the prevalence of NCDs and the advertising of unhealthy food.

The acceptance of countermeasures targeting CDoH was based on three items inquiring about respondents’ attitudes toward the following:

- Restrictions on advertising unhealthy food, similar to the case of tobacco products.
- The exclusion of advertising unhealthy products during sports events.
- The feasibility of fiscal interventions, e.g., a sugar tax, as a preventive intervention in the case of obesity.

#### 2.3.1. European Health Literacy Survey Questionnaire (HLS-EU-Q6)

The HLS-EU-Q6 is a short variant of the questionnaire developed by Sørensen et al. within the European project [36]. The final score reflecting respondents’ health literacy (HL) was calculated as described above [37]. The ‘difficult to say/not applicable’ responses are treated as missing values. A total score of ≤2 indicates inadequate, one from >2 to 3 indicates problematic, and one of >3 indicates sufficient HL. In this analysis, HL was used as a categorical variable assuming four categories. In case the level of HL could not be established because of too many (more than one of the six items) missing values, the category of ‘undetermined’ was introduced.

#### 2.3.2. Short Food Literacy Questionnaire (SFLQ)

The Polish version of the SFLQ consists of 11 items [38]. Respondents can select 4–6 options when answering. The total food literacy (FL) score, calculated as a sum of individual scores after converting responses to numerical values, can range from 6 to 48.

#### 2.3.3. e-Health Literacy Scale (eHEALS)

The eHEALS is frequently used to measure the digital HL of respondents. It was initially developed by Norman and Skinner [35]. The version adapted to Polish was published by Duplaga et al. in 2019 [34]. Respondents respond to 8 items, selecting an option on a 5-item Likert scale from ‘decidedly disagree’ to ‘decidedly agree’. The total score reflecting e-health literacy (eHL) may range from 8 to 40.

#### 2.3.4. Sociodemographic and Economic Characteristics

The determinants of the awareness and the acceptance of restrictions on CDoH analyzed in this study included, in addition to the measurement of the three HL types, sociodemographic and economic variables. They included gender (female or male), age (as a continuous variable), the level of education (four categories: lower than secondary, secondary or post-secondary non-university, bachelor’s, or master’s levels), vocational status (employee, self-employed or farmer, retired or on disability pension, student of high school or university, unemployed, or other status), marital status (married, in partnership, single, or separated/divorced/widowed), and the income per household member (five categories: up to PLN 1500, PLN 1501–3000, PLN 3001–5000, above PLN 5000, or refusal to respond). We also included a variable based on a question about the prevalence of chronic diseases in the analysis.

### 2.4. Statistical Analysis

The IBM SPSS Statistics v.29 package (IBM Corp., Armonk, NY, USA) was used to perform the statistical analysis. For categorical variables, absolute and relative frequencies were calculated. The means and standard deviation (SD) were obtained for continuous numerical variables.

The variables reflecting the awareness of CDoH and the acceptance of restrictions to advertising or fiscal intervention were dichotomized. Responses from ‘decidedly disagree’ to ‘rather disagree’ and ‘neither disagree nor agree’ were collapsed to a value of 0 and, in turn, responses from ‘rather agree’ to ‘decidedly agree’ to a value of 1. These variables were applied as dependent variables in multivariable logistic regression models. In each model, HL, eHL, FL, age, gender, level of education, vocational status, marital status, monthly net income per household member, and the prevalence of chronic diseases were included as independent variables.

Multicollinearity was tested before regression models were developed. The Hosmer and Lemeshow chi2 test and the Nagelkerke R2 were calculated. *p*-values, odds ratios (ORs), and 95% confidence intervals (95% CIs) were reported for the independent variables included in the regression models. *p*-values of lower than 0.05 were deemed to be significant.

## 3. Results

### 3.1. Characteristics of the Study Group

The mean age (SD) in the study group was 40.00 (12.80) years, the mean HL was 2.44 (0.44), the mean eHL was 29.49 (5.02), and the mean FL was 31.38 (6.63). Women made up 50.80% of the study group, residents of rural areas made up 37.65% (*n* = 756), and urban areas of >200,000 inhabitants made up 20.62% (*n* = 414). Respondents with at least one chronic disease made up 43.58% (875). The categorization of the variable reflecting the level of HL revealed that respondents with inadequate HL made up 10.27%, those with problematic HL made up 59.28%, those with sufficient HL made up 9.16%, and those with undetermined HL made up 21.30%. The detailed characteristics of the study group were published earlier [41].

### 3.2. The Awareness of CDoH

The percentage of respondents who were undecided as to the role of CDoH was approximately 25% in the case of all three items probing this aspect (Table 1). Respondents agreeing to some extent that people’s decisions about food purchasing are too often guided by advertisement made up 61.50%. Those opposing such a statement made up only 13.79%. The association between the prevalence of NCDs and the advertising of unhealthy food was seen by approximately 52.34% of respondents, while 21.26% did not agree. Finally, nearly 61% of study participants saw a relationship between profits generated by the big food industry and harm to society’s health. Those who did not confirm such a relationship made up approximately 16%.

### 3.3. The Acceptance of Countermeasures

In the case of items asking about the acceptance of restrictive measures targeting unhealthy food, the percentage of undecided respondents ranged from 22.91% to 25.15% (Table 1). Interestingly, restrictions on advertising unhealthy products during sports events were supported by as many as 57% of respondents. Only 18.53% of respondents opposed this. More than 51% of respondents agreed with the proposal to impose similar bans on unhealthy food adverts as in the case of tobacco products. Approximately 23% of respondents did not agree with such a proposal. Interestingly, the introduction of a sugar tax as an instrument to counteract the obesity epidemic was only supported by 31% of respondents, while nearly 46% of them did not agree.

### 3.4. Determinants of the Awareness of CDoH

FL and eHL were consistent determinants in the case of the statements exploring the awareness about CDoH in the area of the food industry (Table 2). An increase in the FL score of one was significantly associated with an increased likelihood of agreeing that advertising too often guides purchasing decisions by 3%, confirming the relationship between the prevalence of civilization diseases and advertisements of unhealthy food by 3%, and confirming the relationship between food industry profits and harm to society’s health by 4%. Regarding an increase in the eHL score of one, the relevant likelihood increases were 4%, 4%, and 5%, respectively. HL measured with the HLS-EU-Q6 was a significant predictor of only one variable reflecting the awareness of CDoH. Respondents with a problematic level of HL were more likely to confirm the relationship between civilization diseases and the advertising of unhealthy food (OR, 95% CI: 1.55, 1.09–2.19). Education level was a significant predictor of two variables measuring the awareness of CDoH. Other sociodemographic measures were significantly associated with one of three dependent variables. Students at high schools and universities were more likely to agree about the excessive role of adverts in guiding purchasing decisions (OR, 95% CI: 1.72, 1.11–2.68). Older persons more frequently supported the statement about the relationship between corporate profits and society’s health (OR, 95% CI: 1.02, 1.01–1.03). People from households with the highest net incomes were more prone to disagree with the statement about this relationship than those with the lowest incomes (OR, 95% CI: 0.66, 0.45–0.96).

Finally, chronic diseases predicted respondents’ agreement with the statement about the relationship between NCDs and advertisements of unhealthy food (OR, 95% CI: 1.24, 1.03–1.50) and the statement about the relationship between corporate profits and harm to society’s health (OR, 95% CI: 1.32, 1.08–1.61).

### 3.5. Determinants of One’s Attitude toward Measures to Counteract the Effects of CDoH

Once again, SFL was consistently associated with all three variables measuring the attitude toward countermeasures to CDoH in the area of food production and distribution (Table 3). An increase in the SFL score of one was significantly associated with an increased likelihood of supporting restrictions on advertising unhealthy products during sports events by 6%, general restrictions on advertising unhealthy food products by 4%, and the introduction of a sugar tax by 4%. eHL was a significant determinant of two of the three dependent variables in this cluster: supporting the ban on advertising unhealthy food (OR, 95% CI: 1.03, 1.01–1.05) and the introduction of a ‘sugar tax’ (OR, 95% CI: 1.03, 1.01–1.06).

HL was a significant predictor of two of the three variables assessing the attitudes toward countermeasures against CDoH: the prohibition of advertising unhealthy food and the introduction of a sugar tax. Those with problematic HL were more likely than those with inadequate HL to support both measures (OR, 95% CI: 1.60, 1.12–2.29 and 1.56, 1.05–2.32, respectively). Furthermore, respondents with sufficient HL were 50% more likely to support a ‘sugar tax’ than those with inadequate HL.

Respondents with higher levels of education were more in favor of restrictions to adverts during sports events and general restrictions on unhealthy food advertising (OR, 95% CI for the comparison of the respondents who achieved the highest and lowest levels of education: 1.66, 1.21–2.27, and 1.96, 1.43–2.69, respectively). Similarly, older respondents supported such restrictions more frequently than younger ones (OR, 95% CI: 1.01, 1.00–1.02 and 1.02, 1.01–1.03, respectively) and students more frequently than employees (OR, 95% CI: 1.64, 1.07–2.52 and 1.60, 1.05–2.45, respectively). Males were less likely than females to support restrictions on adverts during sports events (OR, 95% CI: 0.71, 0.59–0.87) and the introduction of a sugar tax (OR, 95% CI: 0.77, 0.63–0.95).

Respondents with chronic diseases supported restricting adverts during sports events and introducing a sugar tax (OR, 95% CI: 1.23, 1.01–1.50 and 1.26, 1.03–1.54, respectively).

## 4. Discussion

In our study, we analyzed how Polish adults perceive the impact of food industry CDoH on the prevalence of NCDs and the introduction of countermeasures to restrict unhealthy food advertising and distribution. Our analysis was based on a set of variables developed based on the literature addressing the topic of CDoH, reflecting the awareness (the impact of adverts on food purchases, the relationship between advertising unhealthy food and the prevalence of non-communicable diseases, and the relationship between big industry profits and harm to society’s health) and acceptance of countermeasures to CDoH’s effects (restrictions on advertising unhealthy products during sports events, general restrictions on advertising unhealthy food, introducing additional taxation on SSBs). We found that both FL and eHL were consistent predictors of the awareness of CDoH and the acceptance of introducing relevant restrictions. Among the sociodemographic factors, the level of education and age were important determinants of the dependent variables. The prevalence of chronic diseases was also significantly associated with most variables, which reflected awareness of CDoH and the attitude toward the proposed restrictions. The respondents suffering from chronic diseases were more aware of the impact of CDoH on health and more frequently supported the introduction of relevant restrictions.

To our knowledge, individual perceptions of CDoH had not been studied or analyzed extensively before this study [1,2,3,4,7]. As mentioned above, the public health community only relatively recently adopted the concept of CDoH, reflecting its growing understanding of the impact of big industries on health [12,42]. Furthermore, health professionals’ emphasis on individual responsibility for the prevalence of long-term medical conditions and the necessity of lifestyle changes in advising patients also slowed the introduction of knowledge about CDoH to public opinion. The trends for blaming obesity on individual decisions have been clearly shown in the international survey performed within the EATWELL project. Respondents recruited from the general population were decidedly more inclined to indicate factors such as the lack of willpower (78.4% of supporting opinions) or striving for immediate satisfaction (71.5% of opinions) as responsible for obesity than high exposure to snack foods in workplaces, shops, and homes (59.5%) or too much unhealthy and fatty food in restaurants and supermarkets (58.4%) [43]. A study performed in 2018 in Australia revealed similar findings [44]. Among the contributing factors to the increased rate of overweight and obesity, 86.1% of respondents indicated poor eating and activity choices and 80.6% indicated parental influence on children’s eating and activity, while only 65.3% pointed to the advertising of unhealthy food and drinks.

It also seems that researchers have been more inclined to work on the broader view, developing complex tools to measure corporate influence on health, and proposing multidimensional indicators or agency components [24,25,26]. Usually, the aspect of society’s perception of CDoH has only been indirectly addressed. In our opinion, the awareness and understanding of CDoH in society is critical for designing and implementing appropriate public health strategies to decrease their harmful impact.

In his editorial from 2012, Hastings posed several important questions related to the harm to public health inflicted by economic systems and the marketing practices of corporations [42]. Why should marketing not be seen as ‘a carefully controlled responsibility’? Furthermore, he also declared that all corporations should be required to show the effect of their marketing on health and welfare. These considerations were included in the concept of CDoH which was thoroughly elaborated in the following years.

The importance of advertising in business practices is not questioned. It seems that subconsciously, we assume that ‘marketing is currently a right taken for granted’ [42]. This is probably why broader audiences are rarely asked if they would consent to restrictions on marketing and advertising products with harmful health effects. The way tobacco restrictions were developed is perceived by public health professionals as an example of a successful implementation of policies to decrease the risk of NCDs. In our study, as many as 52% of respondents would accept similar restrictions on advertising unhealthy food as in the case of smoking products. This is a rather unexpected finding for us as the proposed actions are rather severe and located high on the intervention ladder [45]. A European survey performed in 2011 showed that only 36.1% of respondents in Poland supported restricting unhealthy food advertising [43]. The support for such restrictions spanned from 33.0% in Denmark to 57.1% in Italy. Apparently, people are now more open to policies that seek to reduce risk factors and improve their health, but progress is rather slow. The acceptance of the ban on advertising products with a high sugar content was one of the items covered by the survey conducted by Hagmann et al. [31]. Interestingly, the mean score showing the acceptance of such measures on a scale from 1 to 7 spanned from 4.01 for the German sample to as much as 4.88 for the French-speaking population in Switzerland. Therefore, the finding for the first sample is similar to the Polish one. Reynolds et al. also underlined that public opinion had an overall favorable assessment of nudges and taxes introduced to improve population health [32]. However, these authors simultaneously stressed the differences in acceptance depending on the type of behavior and type of intervention.

People are encouraged to undertake sports as part of a healthy lifestyle. On the other hand, sports events are populated with adverts and campaigns promoting beer, fast food, and other products that exert harmful effects on health. The history of cooperation between large-scale sporting events and corporations goes back to the 1920s [46]. In recent years, the paradox of promoting unhealthy products and alcoholic beverages at sporting events has been commented on by many authors [20]. However, it seems that the public health community is not able to influence the corporate agents and organizations behind sports events to restrict the advertising of unhealthy products. The case of the Fédération Internationale de Football Association (FIFA) overruling a law to prohibit the sale of alcoholic beverages during sporting events for the World Cup in Brazil shows that international organizations and corporations are able to resist national policies [47].

The case of marketing unhealthy products at sports events remains a subject of controversy. Some authors comment that there is still insufficient evidence on the harmful effects of advertisements for beer or other products on health [20]. According to the study performed by McDaniel and Mason, public opinion perceives the advertising of cigarettes and beer differently, agreeing with the restrictions on the first products while accepting the promotion of beer during sporting events [48]. The observation that 57% of respondents agreed about restricting the advertising of unhealthy products during sports events may suggest that developing relevant legislation may now be more welcomed by societies. Even higher support for restrictions on advertising unhealthy food and beverages during sports events, surpassing 70%, was recently reported in Spain [49].

The acceptance of fiscal interventions aimed at decreasing the consumption of unhealthy food, especially products with a high sugar content, has been examined thoroughly. A ‘sugar tax’ has already been introduced in more than 40 countries [50]. Special taxation of sugar-sweetened beverages (SSBs) was introduced in Poland in 2021 [51]. This is still a subject of political debate and conflicting views [52].

Our analysis showed that only 32% of respondents supported the statement that a tax on SSBs is an appropriate measure to counteract the obesity epidemic, and 46% did not agree. Interestingly, a telephone-based survey performed in 2016 among Polish adults showed a very similar distribution of responses to this item: 37.3% supported the introduction of a tax on SSBs, and 40.2% did not [53]. Low acceptance of fiscal intervention may depend on several factors. First, an additional burden on individuals’ personal budgets is rarely seen as an appropriate measure [31]. People also tend to perceive fiscal intervention as intrusive and, as such, it receives less support. Lower acceptance of more intrusive actions focused on health behaviors was reported by Diepeveen et al. based on a broad systematic review [54]. Such an association was also reported later by other authors focusing on interventions to reduce the consumption of unhealthy food products [31,32,33,55,56]. Pettigrew et al. showed that public opinion’s preference for interventions that rely on information provision over more intrusive measures is not specific to high-income countries but is also typical for countries with lower levels of income [56]. Second, public opinion may not be fully aware to what degree an increase in obesity prevalence is associated with the consumption of SSBs, as evidenced by their high global intake [57,58]. Furthermore, the lack of appropriate information campaigns could result in a situation in which society is unaware of the impact of simple fiscal interventions and their benefits to health [59]. Finally, the unclear destination of the income from the tax and uncertainty about whether it is actually used to benefit common health can lead to general skepticism [59]. The analysis performed by Dieteren et al. also showed that the level of acceptance of a fiscal intervention strongly depends on its dimension, e.g., the level of the additional tax [60].

Our study was focused on the CDoH associated with the food industry and we did not make comparisons with other industries that offer products with a harmful impact on health, commonly targeted by researchers. However, it should be noted that previous studies clearly showed diversified attitudes of societies toward interventions aiming at the reduction of the use or consumption of different products. Those targeting tobacco use are commonly significantly more accepted in public opinion than those related to alcohol and food [32,61].

As expected, higher education was associated with better awareness of CDoH’s impact and higher acceptance of restrictive measures. Other authors reported different results, at least in relation to accepting interventions. Petrescu et al. did not see a significant relationship between the level of education and the attitude toward nudging interventions to reduce obesity in either the UK or the USA [33]. Furthermore, no significant relationship was confirmed between education and the acceptance of a set of measures aimed at lowering sugar intake in Swiss [31] or Spanish [49] populations. In turn, Pettigrew et al. found that support for a set of nutrition interventions and policies was positively associated with education level [56].

Our study also confirmed a consistent association between age and dependent variables. In 1990, Jeffery et al. reported higher support for interventions to moderate the use of unhealthy products among older rather than younger respondents [61]. Similarly, as in our study, they also found that women are more likely to support such interventions [61]. Higher support among women and older people for governmental interventions aimed at changing health behaviors was also confirmed in a systematic review published by Diepeveen et al. in 2013 [54]. A recent survey in seven countries showed similar relationships [56]. Higher approval among women than men for interventions to reduce sugar intake has also been described by other authors [31,56]. In the Spanish population, women were observed to have significantly higher acceptance of nearly all analyzed interventions proposed as countermeasures by means of reducing the consumption of unhealthy food and beverages [49].

Our study did not show any differences in attitudes toward advertising or the introduction of a tax on SSBs depending on the income status of the household, and earlier studies provided conflicting results. According to Diepeveen et al., a few identified studies showed that lower-income groups support policies focused on diet and physical activity more than higher-income groups, but another showed the opposite relationship [54]. A study analyzing the acceptance of interventions to reduce sugar intake in the Swiss population did not reveal a significant effect of the level of income [31].

Higher levels of SF and eHL predicted greater CDoH awareness in society and the acceptance of restrictions on advertising and fiscal intervention for unhealthy food. In the case of SF, knowledge and skills regarding retrieving and using information about healthy nutrition and food translate directly into a better understanding of the consequences of consuming highly processed food, sweetened beverages, and alcohol. A positive effect of higher eHL on the awareness of CDoH and acceptance of restrictions may result from higher exposure to diversified sources of information about the activities of big industries.

Unexpectedly, HL was a significant predictor of only one dependent variable used to measure CDoH awareness. This may show a relatively low emphasis on education about CDoH among various society-level interventions targeted at knowledge about health. This is in line with the opinion of Tshekiso, who commented that HL traditionally focuses on an individual’s ability to process health information but neglects the societal and structural forces that determine people’s choices [62]. Furthermore, in the report published under the auspices of the World Health Organization, CDoH is indicated as one of the greatest challenges to the vision of HL [63].

People with higher levels of HL were more likely to support the general prohibition of unhealthy food advertising and a sugar tax than those with the lowest level of HL. The latter findings are in line with our previous study on the determinants of accepting a sugar tax [53]. We must underline that in the current study, the most significant differences were observed between respondents with inadequate and problematic HL and only in one case between those with inadequate and sufficient HL. At this stage, we cannot explain why the latter group did not differ significantly in CDoH awareness and the acceptance of the ban on advertising. We can only speculate that those with a sufficient level of HL believe they are able to make their own decisions about food choices, and they perceive external stimuli as not needed to adhere to health behaviors. Possibly, those with problematic HL who feel uncertain of their knowledge and skills about health expect that some decisions or guidance should be made for them by governments or other external bodies. Assuming that health behaviors may reveal the level of an individual’s HL, we can also look back to earlier studies analyzing the association of such behaviors with attitudes toward policy interventions. According to the systematic review by Diepeveen et al. [54], four out of six studies assessing the acceptability of interventions and an individual’s own diet and physical activity have not found a significant relationship. Two others showed opposite effects of a high BMI on the acceptability of interventions. Interestingly, the survey reported in 2018 by Hagmann et al. [31] revealed that overweight participants and those consuming more SSBs strongly opposed interventions aimed at reducing sugar intake.

Our study showed that respondents suffering from chronic diseases are more aware of the role of CDoH in influencing people’s health. We believe that this relationship may be related to more frequent contact with health professionals and the educational effect resulting from such interactions. General practitioners usually support their patients with chronic medical conditions, e.g., coronary heart disease, diabetes, or obesity, with advice about limiting or ceasing the consumption of certain products.

Respondents with chronic disease were also more likely to accept restrictions on advertising unhealthy products during sports events and the introduction of an SSB tax, but not a general restriction on advertising unhealthy food. Such findings are not fully in line with those reported by other authors. For example, Pettigrew et al. observed, based on a multinational survey, that higher self-rated health is associated with stronger support for nutritional interventions and policies [56].

### Limitations

Our study has several limitations. First, the analysis reported herein is based on a survey performed among Internet users. Internet use is steadily growing in Poland, but the older population suffers from a digital divide. As a result, this population segment was underrepresented in our sample, and we received feedback on CDoH that may not fully represent the entire adult Polish population. Furthermore, Internet users differ from non-users in the range of explored information sources and the perception of corporations’ activities. They are also exposed to more diversified marketing interventions developed by businesses. Finally, the Internet is currently a platform for accessing diversified types of e-services, including purchases of various products.

We used ad hoc measures of the awareness of CDoH and the acceptance of measures to limit their impact on respondents. As these attitudes within societies have not frequently been studied, apart from the acceptance of fiscal interventions targeting products with high sugar content, we treated our study as an attempt to explore a new area and, thus, as preparation for the development of standardized tools that could be used in further research on CDoH.

We also believe that national characteristics and economic systems’ local specificity may drive the perception of CDoH. Therefore, the extrapolation of our results to other populations may be unfeasible.

The limited questionnaire size led to the use of a short version of the HL instrument. This could add to a lower sensitivity of HL measurement among respondents. We must also admit that we did not include in our analysis other variables that could be potentially associated with the awareness of CDoH or the acceptance of relevant countermeasures, e.g., the current health behaviors of the respondents, the perceived effectiveness of an intervention, or the political orientation of respondents [32,33].

In the current paper, we did not address the question of a possible relationship between the awareness of CDoH and the acceptance of the postulated restrictions. We believe these issues deserve a dedicated analysis and report.

As always in the case of studies employing a cross-sectional design, we can only examine associations between variables and cautiously use the results to better understand the causal relationships occurring in the population.

## 5. Conclusions

Today, CDoH are perceived as one of the greatest public health and health promotion challenges. The WHO postulates a significant change in the concept of HL toward a better understanding of social determinants, including CDoH. The results of our study reveal that the awareness of the impact of CDoH is relatively high in Polish society, as more than 50% of Polish adult Internet users associated the consumption of processed food, SSBs, and alcohol with a health risk. They also confirmed that the profits of big food industries are achieved at the expense of society’s health. It may also be encouraging that most respondents supported the introduction of restrictions on advertising unhealthy food. However, the inconsistent role of HL as a determinant of the awareness of CDoH and the proposed measures is concerning. This shows that the proposals to extend the concept of HL to include knowledge on corporate influence may be right. It is also noticeable that FL and eHL are more consistent predictors of attitudes toward CDoH than HL.

The fact that CDoH are drivers of NCDs cannot be ignored, and public health systems should systematically target them. Their observed multidimensional influence, including in the realms of politics and legislation, speaks for remodeling interventions aimed at developing society’s HL and its awareness of the role of CDoH in the modern world. We believe that this is a critical condition for raising support for policies to counteract the harmful effects of CDoH.

The awareness of CDoH in Polish society remains at a relatively low level and requires extensive educational interventions. Mature attitudes toward advertising content should be promoted among citizens. In the face of the epidemic of NCDs, the public health community should consider more restrictive measures aimed at lowering the impact of CDoH. However, their successful implementation requires intensive efforts to raise the awareness of CDoH.

Our study revealed that further research is needed on the relationship between health literacy and CDoH. It should explain whether the lack of a consistent relationship between HL and awareness of CDoH or acceptance of proposed measures is related to the limitations of the applied instrument or if it is an issue of the gaps in health education addressed to society.

Decidedly, research on the relationship between the awareness of CDoH and the acceptance of countermeasures to their influence is also needed. Findings from such research could guide health promotion interventions, decreasing society’s susceptibility to marketing and advertising unhealthy food and other products.

## Figures and Tables

**Table 1:** The distribution of responses to items assessing respondents’ awareness of CDoH and attitudes toward measures decreasing their impact on health (*n* = 2008).

Item	Decidedly Disagree % (*n*)	Disagree % (*n*)	Rather Disagree % (*n*)	Neither Disagree nor Agree % (*n*)	Rather Agree % (*n*)	Agree % (*n*)	Decidedly Agree % (*n*)
The awareness of CDoH in the area of the food industry							
Purchasing food is too often guided by adverts.	2.54 (51)	3.88 (78)	7.37 (148)	24.70 (496)	31.62 (635)	19.07 (383)	10.81 (217)
Civilization diseases, e.g., myocardial infarction, stroke, and obesity, are associated with advertising of unhealthy food.	4.08 (82)	6.18 (124)	11.01 (221)	26.39 (530)	24.10 (484)	18.13 (364)	10.11 (203)
Big industries producing sweets, sweetened soft drinks, highly processed food, and chains offering fast food achieve profits at the expense of society’s health.	2.69 (54)	5.03 (101)	7.87 (158)	23.51 (472)	25.35 (509)	20.77 (417)	14.79 (297)
The acceptance of restrictions on advertising and distribution of unhealthy food products							
Products that have harmful effects on health should not be advertised during sports events.	4.38 (88)	6.92 (139)	7.22 (145)	24.80 (498)	16.43 (330)	19.97 (401)	20.27 (407)
Advertising unhealthy food should be banned, similarly to advertising of tobacco products.	5.03 (101)	7.92 (159)	10.26 (206)	25.15 (505)	19.92 (398)	15.69 (315)	16.14 (324)
Increased taxation of sweetened beverages (a ‘sugar tax’) is an appropriate method of counteracting the obesity epidemic.	19.02 (382)	13.89 (279)	12.90 (259)	22.91 (460)	14.64 (294)	10.01 (201)	6.62 (133)

Abbreviations: CDoH—commercial determinants of health.

**Table 2:** Multivariable logistic regression models for dependent variables reflecting the awareness of CDoH.

Independent Variable	Category	Food Purchases Are too Often Guided by Adverts		Advertising of Unhealthy Food Is Associated with the Prevalence of Civilization Diseases		Big Industry Profits Are at the Expense of Society’s Health	
		OR (95% CI)	*p*	OR (95% CI)	*p*	OR (95% CI)	*p*
HL	Inadequate *						
	Problematic	1.06 (0.74–1.50)	0.757	1.55 (1.09–2.19)	0.014	1.30 (0.91–1.86)	0.154
	Sufficient	1.13 (0.88–1.45)	0.337	1.10 (0.86–1.41)	0.441	1.08 (0.84–1.39)	0.549
	Undetermined	1.38 (0.89–2.12)	0.147	1.16 (0.77–1.74)	0.484	0.77 (0.50–1.17)	0.221
FL		1.03 (1.01–1.05)	0.002	1.03 (1.02–1.05)	< 0.001	1.04 (1.03–1.06)	< 0.001
eHL		1.04 (1.02–1.06)	< 0.001	1.04 (1.02–1.06)	0.001	1.05 (1.02–1.07)	< 0.001
Age		1.00 (0.99–1.01)	0.371	1.00 (0.99–1.01)	0.778	1.02 (1.01–1.03)	0.003
Gender	Female *						
	Male	1.18 (0.97–1.45)	0.095	1.04 (0.85–1.26)	0.726	1.05 (0.86–1.29)	0.608
Education	Lower than sec. *						
	Sec. or post-sec. non-university	1.50 (1.16–1.96)	0.002	1.23 (0.95–1.60)	0.118	1.53 (1.17–1.99)	0.002
	University bachelor’s degree	1.77 (1.24–2.53)	0.002	1.18 (0.83–1.66)	0.354	1.63 (1.14–2.33)	0.007
	University master’s degree	1.60 (1.17–2.18)	0.003	1.35 (0.99–1.83)	0.058	1.96 (1.42–2.69)	< 0.001
Marital status	Married *						
	In partnership	1.20 (0.90–1.60)	0.209	1.08 (0.82–1.42)	0.595	0.93 (0.70–1.23)	0.603
	Single	1.02 (0.79–1.33)	0.853	0.90 (0.70–1.16)	0.413	0.89 (0.68–1.15)	0.359
	Separated/divorced/ Widowed	1.10 (0.81–1.49)	0.551	1.13 (0.84–1.52)	0.422	1.09 (0.80–1.50)	0.573
Vocational status	Employee *						
	Self-employed or farmer	1.10 (0.81–1.48)	0.547	1.27 (0.95–1.70)	0.107	1.07 (0.79–1.45)	0.673
	Retired or on disability pension	0.83 (0.58–1.17)	0.280	0.87 (0.62–1.22)	0.414	0.74 (0.52–1.06)	0.104
	High school or university student	1.72 (1.11–2.68)	0.016	1.05 (0.70–1.60)	0.802	1.39 (0.91–2.13)	0.129
	Unemployed or other	0.85 (0.65–1.11)	0.236	0.89 (0.68–1.16)	0.391	0.90 (0.68–1.18)	0.437
Net income per household member	≤PLN 1500 *						
	PLN 1501–3000	0.94 (0.70–1.25)	0.655	0.84 (0.63–1.11)	0.216	0.74 (0.55–0.999)	0.0497
	PLN 3001–5000	1.03 (0.75–1.43)	0.838	0.97 (0.71–1.34)	0.874	0.77 (0.56–1.08)	0.130
	>PLN 5000	0.86 (0.59–1.25)	0.438	0.90 (0.62–1.29)	0.557	0.66 (0.45–0.96)	0.031
	Refusal to respond	0.94 (0.67–1.32)	0.715	0.85 (0.61–1.19)	0.336	0.73 (0.51–1.03)	0.073
Chronic diseases	No *						
	Yes	1.16 (0.95–1.41)	0.148	1.24 (1.03–1.5)	0.026	1.32 (1.08–1.61)	0.006

Abbreviations: OR—odds ratio, *p*—*p*-value for independent variable, 95% CI—95% confidential interval, *—referential category of variable, CDoH—commercial determinants of health, HL—health literacy, FL—food literacy, eHL—e-health literacy, sec.—secondary, post-sec.—post-secondary, and PLN—Polish zloty.

**Table 3:** Multivariable logistic regression models for dependent variables reflecting attitudes toward countermeasures against CDoH.

Independent Variable	Category	Products with Harmful Health Effects Not Advertised during Sports Events		Advertising of Unhealthy Food Banned, Similar to Tobacco Products		Increased SSB Tax Appropriate to Counteract the Obesity Epidemic	
		OR (95% CI)	*p*	OR (95% CI)	*p*	OR (95% CI)	*p*
HL	Inadequate *						
	Problematic	1.10 (0.77–1.56)	0.612	1.6 (1.12–2.29)	0.009	1.56 (1.05–2.32)	0.028
	Sufficient	0.97 (0.75–1.24)	0.795	1.08 (0.84–1.39)	0.536	1.5 (1.13–1.99)	0.005
	Undetermined	1.21 (0.79–1.86)	0.371	1.04 (0.69–1.57)	0.856	1.41 (0.91–2.17)	0.121
SFL		1.06 (1.04–1.08)	<0.001	1.04 (1.02–1.06)	<0.001	1.04 (1.02–1.06)	<0.001
eHL		1.01 (0.99–1.03)	0.492	1.03 (1.01–1.05)	0.005	1.03 (1.01–1.06)	0.007
Age		1.01 (1.00–1.02)	0.045	1.02 (1.01–1.03)	<0.001	1 (0.99–1.01)	0.546
Gender	Female *						
	Male	0.71 (0.59–0.87)	0.001	0.86 (0.71–1.05)	0.130	0.77 (0.63–0.95)	0.013
Education	Lower than sec. *						
	Sec. or post-sec. non-university	1.59 (1.22–2.07)	0.001	1.25 (0.96–1.63)	0.104	0.84 (0.63–1.11)	0.223
	University bachelor’s degree	1.72 (1.21–2.44)	0.003	1.34 (0.94–1.90)	0.108	0.78 (0.54–1.14)	0.197
	University master’s degree	2.11 (1.54–2.89)	<0.001	1.66 (1.21–2.27)	0.002	0.79 (0.57–1.1)	0.166
Marital status	Married *						
	In partnership	1.03 (0.77–1.36)	0.861	1.04 (0.79–1.38)	0.765	0.9 (0.67–1.22)	0.510
	Single	1.03 (0.80–1.33)	0.835	0.68 (0.52–0.87)	0.003	0.98 (0.74–1.29)	0.871
	Separated/divorced/ widowed	1.09 (0.80–1.49)	0.567	0.91 (0.67–1.23)	0.524	1.15 (0.85–1.57)	0.368
Vocational status	Employee *						
	Self-employed or farmer	0.96 (0.71–1.28)	0.769	1.01 (0.75–1.35)	0.951	0.99 (0.73–1.34)	0.952
	Retired or on disability pension	0.84 (0.59–1.20)	0.338	1.07 (0.75–1.53)	0.693	1.02 (0.71–1.46)	0.916
	High school or university student	1.64 (1.07–2.52)	0.023	1.60 (1.05–2.45)	0.030	0.92 (0.57–1.46)	0.710
	Unemployed or other	0.94 (0.72–1.24)	0.684	0.92 (0.70–1.20)	0.528	0.82 (0.61–1.11)	0.201
Net income per household member	≤PLN 1500 *						
	PLN 1501–3000	0.987 (0.75–1.33)	0.987	0.88 (0.66–1.18)	0.396	0.98 (0.72–1.33)	0.910
	PLN 3001–5000	0.77 (0.56–1.07)	0.120	0.81 (0.59–1.12)	0.199	1.11 (0.79–1.56)	0.534
	>PLN 5000	0.94 (0.65–1.37)	0.751	0.89 (0.61–1.28)	0.522	1.05 (0.71–1.55)	0.812
	Refusal to respond	0.82 (0.58–1.15)	0.246	0.70 (0.50–0.99)	0.042	0.73 (0.5–1.07)	0.112
Chronic disease(s)	No *						
	Yes	1.23 (1.01–1.50)	0.035	1.14 (0.94–1.38)	0.190	1.26 (1.03–1.54)	0.027

Abbreviations: OR—odds ratio, *p*—*p*-value for independent variable, 95% CI—95% confidential interval, *—referential category of variable, CDoH—commercial determinants of health, HL—health literacy, eHL—e-health literacy, sec.—secondary, post-sec.—post-secondary, and PLN—Polish zloty.

## Data Availability

The data are not publicly available due to privacy and ethical restrictions. The authors did not include in the information about this study provided to the participants that public access to the data obtained during the survey may be considered. Access to the data will be granted on a case-by-case basis to a justified request after receiving consent from the Bioethical Committee at Jagiellonian University.
